# Anticholinesterase Activity of Eight Medicinal Plant Species: *In Vitro* and *In Silico* Studies in the Search for Therapeutic Agents against Alzheimer's Disease

**DOI:** 10.1155/2021/9995614

**Published:** 2021-06-25

**Authors:** Md Josim Uddin, Daniela Russo, Md Mahbubur Rahman, Shaikh Bokhtear Uddin, Mohammad A. Halim, Christian Zidorn, Luigi Milella

**Affiliations:** ^1^Pharmazeutisches Institut, Abteilung Pharmazeutische Biologie, Christian-Albrechts-Universität Zu Kiel, Gutenbergstraße 76, Kiel 24118, Germany; ^2^Department of Science, University of Basilicata, Viale Dell'Ateneo Lucano 10, Potenza 85100, Italy; ^3^Spinoff BioActiPlant s.r.l., Department of Science, University of Basilicata, Potenza, Italy; ^4^Division of Infectious Diseases and Division of Computer-Aided Drug Design, The Red-Green Research Centre, BICCB, Tejgaon, Dhaka, Bangladesh; ^5^Department of Botany, University of Chittagong, Chattogram 4331, Bangladesh; ^6^Department of Physical Sciences, University of Arkansas-Fort Smith, Fort Smith, AR, USA

## Abstract

Many Bangladeshi medicinal plants have been used to treat Alzheimer's disease and other neurodegenerative diseases. In the present study, the anticholinesterase effects of eight selected Bangladeshi medicinal plant species were investigated. Species were selected based on the traditional uses against CNS-related diseases. Extracts were prepared using a gentle cold extraction method. *In vitro* cholinesterase inhibitory effects were measured by Ellman's method in 96-well microplates. *Blumea lacera* (Compositae) and *Cyclea barbata* (Menispermaceae) were found to have the highest acetylcholinesterase inhibitory (IC_50,_ 150 ± 11 and 176 ± 14 *µ*g/mL, respectively) and butyrylcholinesterase inhibitory effect (IC_50_, 297 ± 13 and 124 ± 2 *µ*g/mL, respectively). *Cyclea barbata* demonstrated competitive inhibition, where *Blumea lacera* showed an uncompetitive inhibition mode for acetylcholinesterase. *Smilax guianensis* (Smilacaceae) and *Byttneria pilosa* (Malvaceae) were also found to show moderate AChE inhibition (IC_50,_ 205 ± 31 and 221 ± 2 *µ*g/mL, respectively), although no significant BChE inhibitory effect was observed for extracts from these plant species. Among others, *Thunbergia grandiflora* (Acanthaceae) and *Mikania micrantha* (Compositae) were found to display noticeable AChE (IC_50_, 252 ± 22 *µ*g/mL) and BChE (IC_50_, 314 ± 15 *µ*g/mL) inhibitory effects, respectively. Molecular docking experiment suggested that compounds 5-hydroxy-3,6,7,3′,4′-pentamethoxyflavone (BL4) and kaempferol-3-*O*-*α*-L-rhamnopyranosyl-(1⟶6)-*β*-D-glucopyranoside (BL5) from *Blumea lacera* bound stably to the binding groove of the AChE and BChE by hydrogen-bond interactions, respectively. Therefore, these compounds could be candidates for cholinesterase inhibitors. The present findings demonstrated that *Blumea lacera* and *Cyclea barbata* are interesting objects for further studies aiming at future therapeutics for Alzheimer's disease.

## 1. Introduction

Alzheimer's disease (AD) is a devastating neurodegenerative disorder linked with the two most common symptoms, memory dysfunction and cognition impairment. In the neuropathological symptoms of AD, cognitive deficit is consistent with the presence of cholinergic deficit, due to the degeneration or atrophy of cholinergic neurons in the basal forebrain, including senile plaques and neurofibrillary tangles [1]. Acetylcholine (ACh), the brain's important natural neurotransmitter, plays a critical role in forming memories, verbal and logical reasoning, and the ability to concentrate. However, ACh's activity is greatly hindered by both acetylcholinesterase (AChE) and butyrylcholinesterase (BChE) enzymes.

Inhibition of the cholinesterase enzymes (AChE and BChE) can boost ACh levels in several parts of the brain and symptoms associated with the progressive loss of cholinergic function in AD improve [2]. Studies also showed that increased ACh concentration in the brain increases the expression of nicotinic ACh receptors related to cognitive function [3, 4]. This phenomenon might help AD patients generating new memories and remembering the older ones. Thus, AChE and BChE inhibition has been established as a primary therapeutic target based on this “cholinergic hypothesis.”

Pharmacological treatment of AD is largely based on treating symptoms and severity with advanced stages rather than targeting the etiological mechanisms. The currently approved medications for AD are mostly cholinesterase inhibitors, including donepezil, rivastigmine, galantamine, and NMDA antagonist memantine [5]. Among the commonly prescribed AChE inhibitors, only donepezil was approved for the treatment of all stages of AD. However, this drug has some severe side effects, including gastrointestinal disturbance, liver-associated problems, and GIT-related abnormalities [6]. Considering all these limitations, it is worthy to find new lead compounds from different sources, including plant-derived natural products.

**Figure 1:**
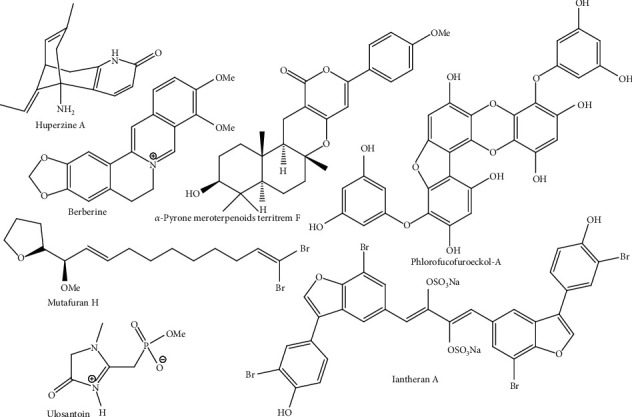
Structures of some representative lead inhibitors against AChE and BChE based on their IC_50_ values (<1 *µ*M).

Natural products have already proven to be promising sources of useful acetylcholinesterase (AChE) inhibitors [7]. The currently approved drugs for AD, galantamine and rivastigmine, are plant-derived alkaloids, which offer symptomatic relief from AD [8]. In addition to these approved natural products, many potent cholinesterase inhibitors have been reported in literature [7]. Some reported potent cholinesterase inhibitors are nevertheless presented in Figure 1.

Our study is an attempt to identify and compare potential candidates from the following eight selected plant species (family affiliations of all species in Table 1) *Blumea lacera* (Burm.f.) DC.*, Byttneria pilosa* Roxb.*, Clerodendrum infortunatum* L.*, Cyclea barbata* Miers*, Mikania micrantha* Kunth, *Smilax guianensis* Vitman*, Spermacoce articularis* L.f., and *Thunbergia grandiflora* Roxb. for anticholinesterase activity in rejuvenating and improving the memory and cognitive function. A study of ethnopharmacological background of the selected species is presented in Table 1.

## 2. Materials and Methods

### 2.1. Chemicals

Acetylcholinesterase (AChE) from electric eel (*Electrophorus electricus*) (type VI-s, lyophilized powder), acetylthiocholine iodide (ATCI), butyrylcholinesterase (BChE) from equine serum (lyophilized powder), butyrylthiocholine iodide (BTCI), and 5,5′-dithio-bis-(2-nitrobenzoic acid) (DTNB) were purchased from Sigma-Aldrich (St. Louis, MO, USA). Trizma hydrochloride (Tris-HCl) and bovine serum albumin (BSA) were obtained from Sigma-Aldrich (Steinheim, Germany). Deionized water was produced using a Milli-Q water purification system (Millipore, Bedford, MA, USA).

### 2.2. Plant Materials

All samples, except *Clerodendrum infortunatum*, were collected from Chittagong Hill Tracts, Bangladesh. The plant materials were certified by one of the authors, Taxonomist Prof. Dr. Sheikh Bokhtear Uddin. All voucher specimens have been deposited in the Department of Botany, University of Chittagong (Supplementary Material, Figure S1). The leaves of *Clerodendrum infortunatum* were collected from Pabna, Bangladesh, and authenticated by Prof. Dr. A.H.M. Mahbubur Rahman, Department of Botany, University of Rajshahi. Botanical names along with accession numbers are presented in Table 1 (Supplementary Material, Figure S1).

### 2.3. Extraction of Plant Materials

Freshly collected plant leaves were dried in the shade and then pulverized. To prepare the extract, 10 g of each plant part was extracted in 50 mL solvent employing the cold extraction method as described in Table 1, at room temperature. Solvent polarity has been selected on the nature of phytochemicals present in the plant species [17]. Filtered extracts were dried in a rotary evaporator under reduced pressure at 35°C temperature.

### 2.4. Determination of Cholinesterase (AChE/BChE) Inhibitory Activities

ChE inhibitory activity was measured based on Ellman's method [18] as reported in a previous study [19]. In the inhibition of AChE activity, the enzyme hydrolyzes the substrate acetylthiocholine resulting in the product thiocholine, which reacts with Ellman's reagent (DTNB) to produce 2-nitrobenzoate-5-mercapto-thiocholine and 5-thio-2-nitrobenzoate, detectable at 405 nm. In this assay, 25 *μ*L of acetylthiocholine iodide (5 mM), 125 *μ*L of DTNB (3 mM), 50 *μ*L of buffer B (50 mM Tris-HCl, pH 8 containing 0.1% BSA), and 25 *μ*L of each test extract solution at the different concentrations or negative control (25% DMSO in MeOH) were mixed and incubated for 10 min at 37°C. The reaction was started by adding 25 *μ*L of 0.05 U/mL AChE. The absorbance was measured at 405 nm, and the reaction rates were calculated by MARS Data analysis software (SPECTROStar NANO, BMG Labtech, Germany). Estimation of BChE inhibition was performed in a similar way described above using 25 *μ*L of 5 mM butyrylthiocholine iodide as substrate and 0.05 U/mL of BChE as an enzyme. Galantamine was used as a positive control for both enzymes. Three independent assays were performed in triplicate at different concentrations.

### 2.5. Mode of Inhibition and Kinetic Parameters

To investigate the type of inhibition of the effective extracts, the enzyme activity was determined in the presence of increasing concentrations of substrate ATCI (0.16–20 mM) and the absence or presence of two/three concentrations of each extract. The analysis of the type of inhibition of AChE activity was determined by the Lineweaver-Burk (LB) plot, whereas the kinetic parameters km and Vmax were obtained by curve fitting according to the classical Michaelis-Menten equation by using GraphPad Prism version 6.

### 2.6. Molecular Docking Protocol

The crystal structure of cholinesterase enzymes has been collected from RCSB PDB (rcsb.org) [20]. PDB ID 4EY7 [21] and 4AQD [22] were assigned for AChE and BChE, respectively. The proteins were prepared in Swiss PDB Viewer [23] and PyMOL.

**Figure 2:**
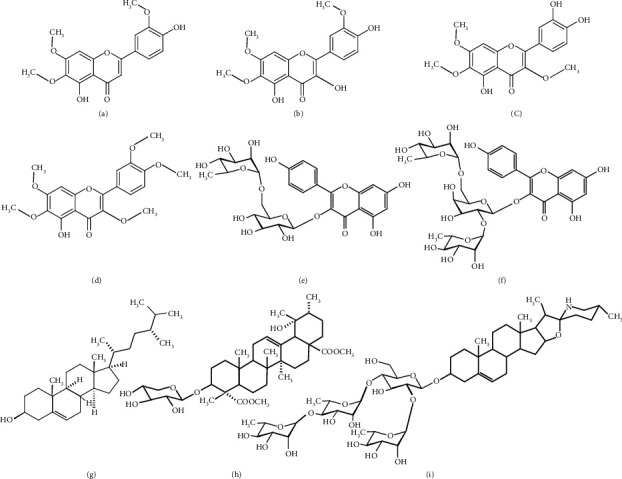
The selected compounds isolated from leaves of *Blumea lacera* for molecular docking with AChE and BChE. (a) 5,4′-dihydroxy 6,7,3′-trimethoxyflavone (**BL1**), (b) 3,5,4′-trihydroxy 6,7,3′-trimethoxyflavone (**BL2**), (c) 5,3′,4′-trihydroxy 3,6,7-trimethoxyflavone (**BL3**), (d) 5-hydroxy 3,6,7,3′,4′-pentamethoxyflavone (**BL4**), (e) Kaempferol-3-*O-α*-L-rhamnopyranosyl-(1⟶6)-*β*-D-glucopyranoside (**BL5**), (f) Kaempferol-3-*O*-(2″,6″-di-*O*-*α*-L-rhamnopyranosyl)-*β*-D-glucopyranoside (**BL6**), (g) Campesterol (**BL7**), (h) 19*α*-hydroxyurs-12-ene-24,28-dioate 3-*O*-*β*-D- xylopyranoside (**BL8**), (i) 25*R*)-3*β*-{*O*-*β*-D-glucopyranosyl-(1⟶ 4)-*O*-*α*-L-rhamnopyranosyl-(1⟶ 4)-[*O*-*α*-L-rhamnopyranosyl-(1⟶ 2)]-*α*-L-rhamnopyranosyl}-22*α*N-spirosol-5-ene (**BL9**).

Nine compounds (Figure 2) from *Blumea lacera* [24–29] were selected for molecular docking study considering their chemical nature and action on cholinesterase [30, 31]. The structures were drawn in ChemDraw and Chem3D. The geometry optimization of all structures was carried out by the semiempirical PM6 method in Gaussian 09 [32]. The optimized structures were collected in PDB format suitable for molecular docking. The molecular docking of all compounds against AChE and BChE was performed in PyRx Virtual Screening Tool (Version 0.8) [33]. The compounds were saved in AutoDock ligand format (PDBQT) before docking. The grid box center and dimensions were set accordingly to cover the substrate-binding sites of the protein. For AChE, the grid box center was reserved at *X* = −2.0303, *Y* = 38.0503, and *Z* = 29.9752, and the dimension (angstrom) was *X* = 42.2467, *Y* = 50.5623, and *Z* = 41.3235. In case of BChE, the grid box center was fixed at *X* = 1.6818, *Y* = −1.3768, and *Z* = −10.0914 and the dimension (angstrom) was *X* = 39.1883, *Y* = 39.4042, and *Z* = 33.0087. The docking process was performed in triplicate for every ligand and the average of the binding affinities has been presented. Furthermore, the extracted ligand from the crystal structure was docked with the optimized protein following the same protocol, which validated our docking protocol [34]. The docked ligand-protein complexes were visualized in BIOVIA Discovery Studio (Version 4.5) to detect the noncovalent interactions.

### 2.7. Statistical Analysis

Data are given as the mean ± SD (*n* = 3). IC_50_ values were calculated via nonlinear regression analysis using GraphPad Prism v. 6.0 (GraphPad Software Inc., USA). One-way analysis of variance (ANOVA) and Tukey test were used to compare means among extracts; an observation was considered statistically significant if the *p* value is less than 0.05 (*p* < 0.05).

## 3. Results and Discussion

**Figure 3:**
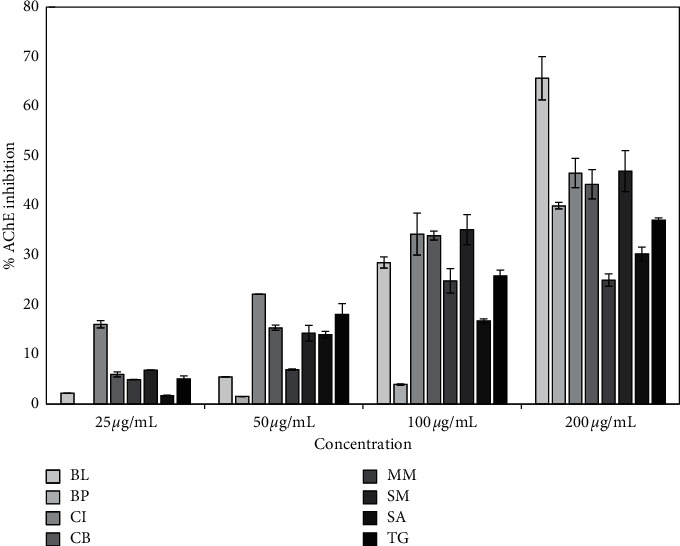
Inhibition of AChE activity of plant extracts. Column represents the inhibition percentage obtained with *Blumea lacera* (BL), *Byttneria pilosa* (BP), *Clerodendrum infortunatum* (CI), *Cyclea barbata* (CB), *Mikania micrantha* (MM), *Smilax macrophylla* (SM), *Spermacoce articularis* (SA), and *Thunbergia grandiflora* (TG). IC_50_ (*µ*g/mL) of positive control galantamine for AChE inhibition assay was 0.92 ± 0.02 *µ*g/mL. Mean values of three independent experiments have been plotted.

The extracts of eight plant species used as herbal medicine in different disorders were tested for AChE and BChE inhibitory activity using Ellman's method. The results are shown in Table 2, representing the IC_50_ values for the eight extracts prepared. The lowest IC_50_ AChE inhibitory activity was found for *Blumea lacera* followed by *Cyclea barbata*, *Smilax guianensis*, *Byttneria pilosa*, and *Thunbergia grandiflora* displayed values of 150 ± 11, 176 ± 14, 205 ± 30, 221 ± 2, and 252 ± 22 *µ*g/mL, respectively.  Figure 3 displays the activity of extracts at various concentrations, where AChE inhibition increases in a dose-dependent manner. Galantamine was used as a standard AChE inhibitor showing IC_50_ of 0.92 ± 0.02 *µ*g/mL (2.49 ± 0.05 *µ*M).

**Figure 4:**
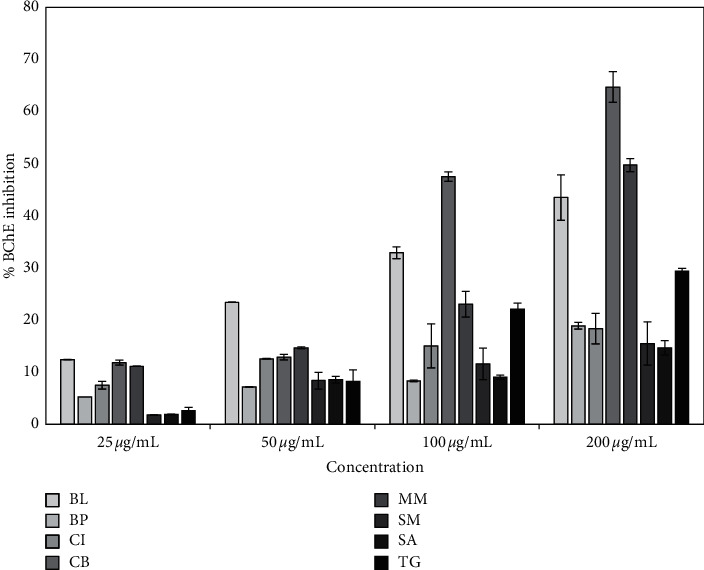
BChE inhibition activity of plant extracts. Column represents the inhibition percentage obtained with *Blumea lacera* (BL), *Byttneria pilosa* (BP), *Clerodendrum infortunatum* (CI), *Cyclea barbata* (CB), *Mikania micrantha* (MM), *Smilax guianensis* (SG), *Spermacoce articularis* (SA), and *Thunbergia grandiflora* (TG). IC_50_ (*µ*g/mL) of positive control galantamine for BChE inhibition assay was 5.97 ± 0.97 *µ*g/mL. Mean values of three independent experiments have been plotted.

In the BChE inhibitory activity, *Cyclea barbata* extract showed the highest inhibition against BChE followed by *Mikania micrantha*, *Blumea lacera*, and *Byttneria pilosa* with their IC_50_ values of 124 ± 2, 216 ± 24, 297 ± 13, 358 ± 32, and 536 ± 23 *µ*g/mL, respectively (Table 2). The IC_50_ value of reference standard galantamine for BChE assay was 5.97 ± 0.97 *µ*g/mL (16.2 ± 2.6 *µ*M). The inhibition of BChE is also increasing corresponding to the concentration of extracts (Figure 4). Our results demonstrated a dual activity of *Blumea lacera*, *Cyclea barbata*, and *Byttneria pilosa* extracts. Since AChE is mainly located in the central nervous system (CNS) and BChE is more abundant in the peripheral system, the potency toward both ChE found in the extracts is of paramount importance. These findings could activate mostly the central as well as peripheral cholinergic transmission to improve the mental abilities of AD patients. Among the eight screened extracts, *Blumea lacera*, *Cyclea barbata*, *Smilax guianensis*, and *Byttneria pilosa* were selected as efficacious candidates as sources of strong ChE (AChE and BChE) inhibitors. Due to the multifactorial pathogenesis of AD, multitargeted drugs will be preferred as the effective therapeutic strategy against AD.

*Blumea lacera*, a widely distributed herb in Bangladesh, is locally known as Kukursunga or Shimura. *B. lacera* is used to cure spasms, fever, and bronchitis and alleviate burning sensation [9]. Leaves of the plant are astringent, stimulant, anthelmintic, antiscorbutic, and diuretic [9, 27] and they are also used as antidysenteric and anti-inflammatory remedies [9]. Acetone extract of leaves of *B. lacera* showed an IC_50_ value of 150 ± 11 and 297 ± 13 *μ*g/mL for AChE and BChE inhibition, respectively; efficacy of these leaves on enhancing cognitive functions might be due to the presence of terpenoids [35]. Acetone-water (70 : 30) extract of *B. lacera* displayed lower activity (IC_50_ value of 197 and 1040 *µ*g/mL for AChE and BChE inhibition, respectively) compared with acetone extract.

*Cyclea barbata* is a tropical indigenous plant widely distributed in Asia, especially in Bangladesh. This climber is traditionally used against various diseases such as headache, epilepsy, allergy, asthma, lipoma, tetanus, and throat sore [9]. Acetone-water (70 : 30) extract of *Cyclea barbata* exhibited IC_50_ value of 176 ± 14 and 124 ± 2 *µ*g/mL for AChE and BChE inhibition, respectively, which might be owing to its high alkaloid contents [36].

*Smilax guianensis,* locally known as Kumarika, is believed to possess CNS-modulating properties and is used in many herbal formulations. *S. guianensis* is also used in the treatment of epilepsy, venereal and skin diseases, fever, swellings, sores, and abscesses [9, 37]. Young stem is used to improve memory loss. The root is used for the management of rheumatism and pain in the lower extremities [15, 38]. Dried leaves of *S. guianensis* methanolic extract inhibited AChE with an IC_50_ value of 205 ± 31 *μ*g/mL which might be due to the presence of steroidal saponin glycosides [39], while no significant inhibition on BChE was observed.

*Byttneria pilosa*, a large woody climber, is widely distributed in Chittagong, Bangladesh. Leaves and stems of this species are usually used to treat snakebite by the traditional healers and indigenous people in Chittagong hill tracts, Bangladesh. It is also used to cure scabies, bone fracture, and elephantiasis [9, 12, 13]. Methanolic extract of *B. pilosa* leaves revealed its AChE and BChE inhibitory potentials with an IC_50_ value of 221 ± 2 and 536 ± 23 *μ*g/mL, respectively, because of the presence of favorable anticholinesterase natural products such as alkaloid, saponin, and terpenoid [13].

Both acetylcholinesterase and butyrylcholinesterase are the key enzymes in the cholinergic nervous system. Therapies designed to reverse the cholinergic deficit in AD are mostly based on inhibitors of AChE. Several studies revealed that cholinesterase inhibitors could act on multiple therapeutic targets such as preventing the formation of *β*-amyloid plaques, antioxidant activity, and modulation of APP processing [40]. Moreover, AChE inhibition is also considered a promising therapeutic strategy for other types of dementia, myasthenia gravis, glaucoma, and Parkinson's disease [41]. Nonetheless, there is still a need to explore the nature of newer potent and long-lasting ChE inhibitors with minimal side effects. Natural products have already been proven to be promising sources of useful ChE inhibitors [7, 42]. Bioassay-guided approaches have studied many plants for the search of new AChE inhibitors with lower toxicity and higher CNS penetration.

**Figure 5:**
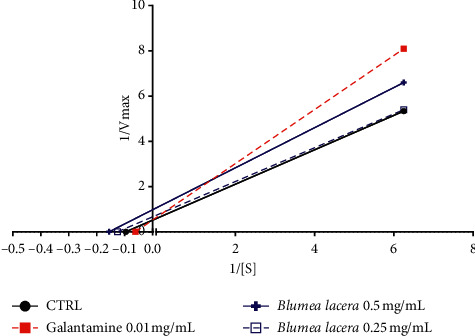
Lineweaver-Burk plot in the absence (control) and presence of inhibitors (galantamine and acetone extract of *Blumea lacera*).

**Figure 6:**
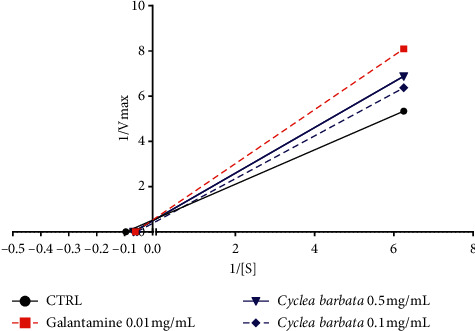
Lineweaver-Burk plot in the absence (control) and presence of inhibitors (galantamine and *Cyclea barbata*).

The kinetic study revealed the potential mechanism of enzyme inhibition. The relationship between substrate concentration and reaction velocity was in good agreement with Michaelis-Menten kinetics. In absence of inhibitors, the km value of the substrate ATCI was 10.4 ± 1.8 mM, and the Vmax was 12.4 ± 1.1 mM/min for the electric eel acetylcholinesterase. Galantamine was used as positive control showing a competitive inhibition. The extracts *Blumea lacera*, *Byttneria pilosa,* and *Smilax guianensis* showed uncompetitive inhibition in which inhibitor only binds to enzyme-substrate complex (Table 3).  Figure 5 represents the enzyme kinetics of *Blumea lacera*. In the uncompetitive pattern, both Vmax and km decrease, and the kinetic pattern produced parallel lines with increasing extract concentration. The mode of inhibition of *Cyclea barbata* seemed to be ambiguous at lower concentration while Vmax and km both increased, whereas at higher concentration, it reflected competitive inhibition (km increased and Vmax remained unchanged) (Figure 6). These types of inhibition could be due to the presence of various phytochemicals typical in medicinal plants. The kinetic study against BChE demonstrated an ambiguous inhibition mode.

*In silico* molecular docking is an efficient tool in recent structure-based drug design. In this anticholinesterase screening study, *Blumea lacera* exhibited the highest inhibition among the eight plant species. Here, the docking study was conducted to identify the responsible compounds from *Blumea lacera* on anticholinesterase potential. However, the binding affinity and binding pose of selected compounds (Figure 2) with the AChE and BChE could be considered as proof of our claimed biological outcome. All the ligands showed good binding affinity where some of them interacted with the catalytic site of proteins (Table 4). BL1, BL2, and BL7 demonstrated the highest binding affinity (kcal/mol) with AChE, whereas BL5, BL6, and BL8 reflected the highest binding affinity with BChE.

**Figure 7:**
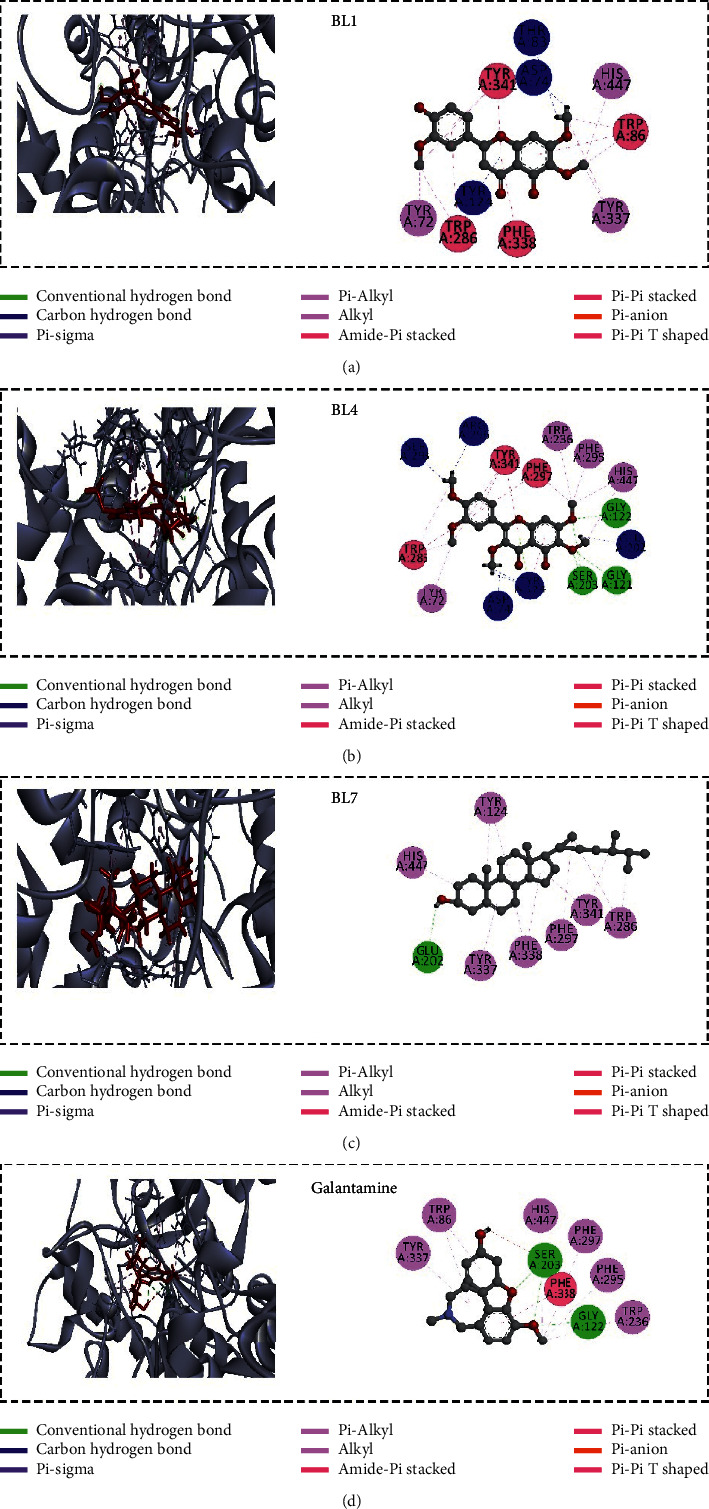
Noncovalent interactions (2D and 3D) of the top three ligands from *Blumea lacera* with AChE (PDB ID 4EY7). In the 3D figure, ligand molecule is represented as red and protein is dark silver (pose predicted by AutoDock Vina). The redocked structure of the native ligand (donepezil) binds to the protein with an RMSD of 0.004 Å. This lower RMSD value validates the docking protocol.

**Figure 8:**
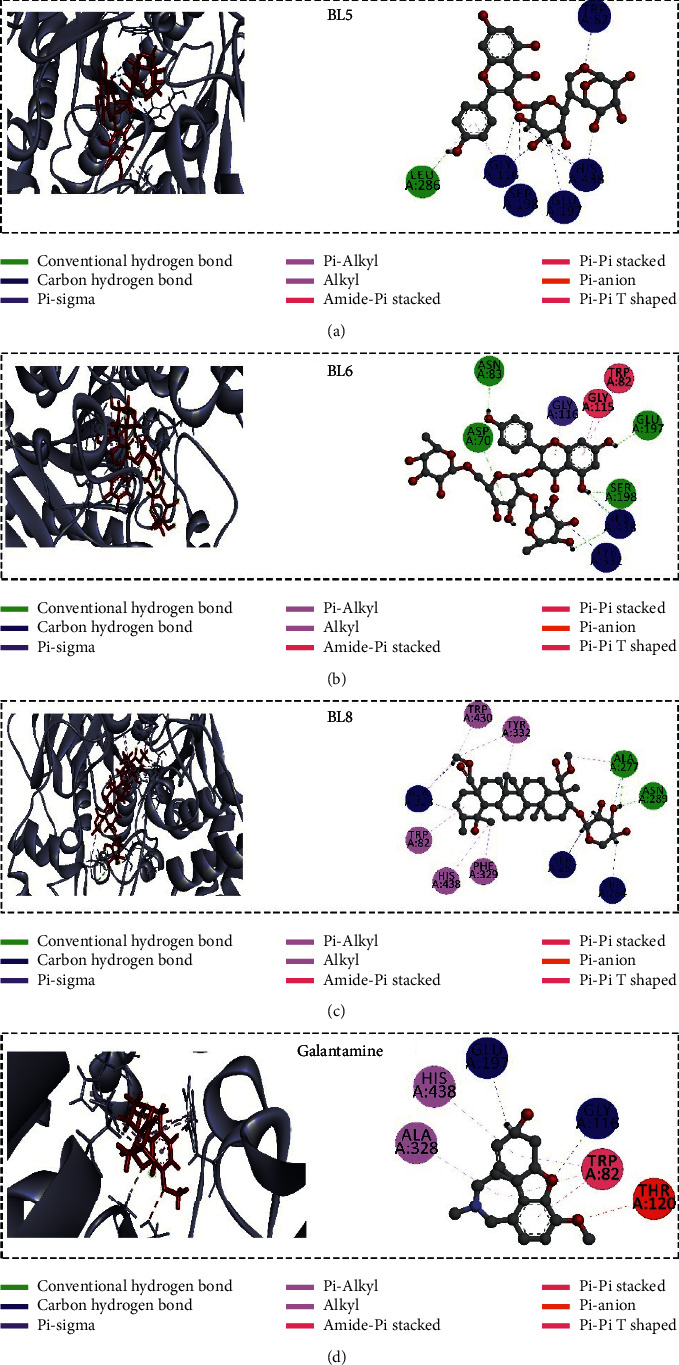
Noncovalent interactions (2D and 3D) of the top three ligands from *Blumea lacera* with BChE (PDB ID 4AQD). In the 3D figure, the ligand molecule and protein are shown in red and dark silver, respectively (pose predicted by AutoDock Vina). The redocked structure of the native ligand (donepezil) binds to the protein with an RMSD of 0.004 Å. This lower RMSD value validates the docking protocol.

Ligands' interaction with the catalytic triad residues of acetylcholinesterase is essential to inhibit enzymatic activity. We analyzed all the ligands binding poses to investigate the interaction of the ligands with the AChE. The majority of the ligands did not interact with the catalytic triad residues of AChE that validated the uncompetitive inhibition mode in the experimental result (Figure 5; Supplementary Material, Figure S2) of *Blumea lacera*. Three out of nine compounds interact with both catalytic and peripheral anionic site (PAS) residues of AChE (Figure 7). All ligands except BL9 interact with the catalytic site residues of BChE (Figure 8; Supplementary Material, Figure S3).

The active site of both AChE and BChE consists of two subsites [43, 44]. The esteratic site (also called catalytic triad) of human AChE consists of three essential amino acids Ser203, Glu334 and, His447, while the PAS is composed of five residues Tyr72, Asp74, Tyr124, Trp286, and Tyr341. The catalytic triad of human BChE is comprised of Ser198, His438, and Glu325, whereas the PAS contains Asp70, Phe329, Trp82, Tyr128, and Tyr332 [45–48]. The binding of a ligand highly regulates the cholinesterase enzyme inhibition at the catalytic site. The aromatic ring in the ligand forms a few pi-alkyl, pi-pi, pi-pi T-shaped interactions with the amino acid residues of the protein. The 5,4′-dihydroxy-6,7,3′-trimethoxyflavone (BL1), 5-hydroxy-3,6,7,3′,4′-pentamethoxyflavone (BL4), and campesterol (BL7) interact with the catalytic residues Ser203 and His447 of AChE reflecting the competitive inhibition (Figure 7). BL1 and BL4 interact with most of the peripheral site residues of AChE, while BL7 interacts only with Tyr124 and Tyr341. Despite having a high binding affinity, 19*α*-hydroxyurs-12-ene-24,28-dioate 3-*O*-*β*-D-xylopyranoside (BL2) does not interact with the PAS and catalytic triad residues. BL1, BL4, and BL7 displayed 16, 16, and 12 hydrophobic interactions as well as three, ten, and one hydrogen bonds with AChE, respectively (Table S1). Many agents could prevent the formation of amyloidogenic protein through blocking the peripheral binding site of AChE and offers additional therapeutic benefits besides the inhibitory activity [49]. BL1 and BL4 acted as a dual-binding agent, blocking both the peripheral and the catalytic binding sites of AChE simultaneously, and could offer additional benefits in AD management.

All compounds, except BL9, exhibited a hydrogen bond with the catalytic site of BChE. The hydroxyl groups in the ligands' side chain have a crucial role in ligand-protein interactions by forming a hydrogen bond with the protein residues. Kaempferol 3-*O*-*α*-L-rhamnopyranosyl-(1⟶6)-*β*-D-glucopyranoside (BL5) formed a hydrogen bond with Ser198 and His438 with side-chain oxygen of BChE. Kaempferol 3-*O*-(2″,6″-di-*O*-*α*-L-rhamnopyranosyl)-*β*-D-glucopyranoside (BL6) interacted with the catalytic residues and PAS residues with side-chain oxygen and hydrogen atoms, forming hydrogen bonds with Asp70, Tyr332, Ser198, and His438 (mixed inhibition). BL8 forms hydrogen bonds with Ala277, Asn289, Ser287, Ala328, and Thr284. BL5, BL6, and BL8 formed 9, 7, and 6 hydrogen bonds with BChE, respectively (Table S2). The methyl groups in the side chain of the ligands play a pivotal role in developing pi-alkyl interaction with the amino residues of the protein. Moreover, BChE has the ability to accommodate bulkier compounds compared to AChE because the active site of BChE has many aromatic residues replaced by residues with aliphatic side chains, such as leucine (Leu) and valine (Val) [49]. Due to space availability, BL5, BL6, and BL8 were able to accommodate and docked completely into the base of the active site and held in place by the hydrogen bond with Ser198 and His438. BL9 might not reach the peripheral site or the catalytic site due to its large molecular structure.

The chemical structure of natural compounds reflects their binding affinity as well as biological activity. It is noted that hydroxylation improves the inhibitory activities of flavonoids against ChE, while methoxylation may decrease or increase these activities [50]. In docking study, it has been observed that the methoxylation of flavonols decreased the binding affinity for ChE; on the other hand, glucose substitution decreased the binding affinity toward AChE but enhanced the affinity for BChE. In case of triterpenoids, glucosylation attenuated the binding affinity for AChE, while binding affinity has been increased against BChE.

## 4. Conclusion

The various traditionally used plant extracts were screened for cholinesterase (AChE and BChE) inhibition assay. The currently approved therapies for AD are based on improving cholinergic transmission via the inhibition of ChE which only provide fair improvement in memory and cognitive functions. This study shows that, out of eight tested species, the leaves of *Blumea lacera, Cyclea barbata*, *Smilax guianensis*, and *Byttneria pilosa* can be selected as promising sources of effective ChE inhibitors. The cholinesterase inhibitory potential of *Blumea lacera* has also been established by molecular docking studies. Since *Blumea lacera* and *Cyclea barbata* extracts can act on both *in vitro* AChE and BChE at a bit higher dose compared to galantamine, further evaluation is required to identify the active ingredients and assess their safety and bioavailability in animal models. With AD being a multifaceted neurodegenerative disease, other targets must be considered for future investigations on these extracts or single compounds along with pharmacokinetic, toxicity, and compound stability studies to establish dose-activity/toxicity relationship and any side effects for potential clinical application.

## Figures and Tables

**Table 1:** Details of the plant species used in this study along with its traditional uses.

Botanical name	Family	Common name	Local name (tribal name)	Parts used	Habitat	Solvent used for extraction	Traditional uses	Accession number
*Blumea lacera* (Burm.f.) DC.	Compositae	Lettuce-Leaf Blumea	Kukursunga, shialmutra	Leaves	Herb	Acetone	Neurological disorder [10], inflammation [11], stimulant, spasm, fever, bronchitis, burning sensation, and dysentery [9]	BL-07052016-7842
*Byttneria pilosa* Roxb.	Malvaceae	Not known	Harjora	Leaves	Climber	Methanol	Antidote of snake poisoning [12], bone fracture, scabies, and elephantiasis [13]	BP-10122013-10578
*Clerodendrum infortunatum* L. (syn.: *Clerodendrum viscosum*)	Lamiaceae	Glory bower	Bhat	Leaves	Undershrub	Acetone	Paralysis [14], snakebite [12], tonic, pain, tumor, skin diseases, cough, and jaundice [9]	CV-20180321-04
*Cyclea barbata* Miers	Menispermaceae	Green grass jelly plant	Patalpur (Chakma, Tanchangya)	Leaves	Shrub	Acetone-water (70 : 30)	Headache, epilepsy, allergy, asthma, lipoma, tetanus, and throat sore [9]	CB-07052015-4974
*Mikania micrantha* Kunth	Compositae	Bitter vine	Assam lata	Leaves	Climber	Methanol	Snakebite [12], ulcer, hemorrhage, and wound [9]	MM-03102016-4985
*Smilax guianensis* Vitman (*Smilax macrophylla* Roxb.)	Smilacaceae	Indian sarsaparilla	Kumarilata, Kumarika	Leaves	Climber	Methanol	Memory loss [15], pain, venereal diseases, sores, abscess, rheumatism, and gonorrhea [9]	SQ-04092016-5610
*Spermacoce articularis* L.f. (*Borreria articularis* F.N. Will.)	Rubiaceae	Shaggy buttonweed	Madnabata kadu	Leaves	Herb	Methanol	Headache [16], inflammation, blindness, earache, and spleen complaints [9]	SA-04092016-5632
*Thunbergia grandiflora* Roxb.	Acanthaceae	Bengal clockvine	Nillata	Leaves	Climber	Acetone-water (70 : 30)	Hysteria, dysentery, cataract, diabetes, gout, hydrocele, and rheumatism [14]	TG-04092016-5608

**Table 2:** Anticholinesterase inhibition (IC_50_, *µ*g/mL) of eight plant extracts and galantamine (positive control).

Species name	AChE inhibition assay	BChE inhibition assay
	IC_50_ (*µ*g/mL)	IC_50_ (*µ*g/mL)
*Blumea lacera* (Burm.f.) DC.	150 ± 11^a^	297 ± 13^c^
*Byttneria pilosa* Roxb.	221 ± 2^a,b^	536 ± 23^d^
*Clerodendrum infortunatum* L.	598 ± 50^d^	971 ± 77^e^
*Cyclea barbata* Miers	176 ± 14^a^	124 ± 2^a^
*Mikania micrantha* Kunth	314 ± 15^b^	216 ± 25^b^
*Smilax guianensis* Vitman	205 ± 31^a^	>1000^f^
*Spermacoce articularis* L.f.	460 ± 126^c^	>1000^f^
*Thunbergia grandiflora* Roxb.	252 ± 22^a,b^	576 ± 64^d^
Galantamine	0.92 ± 0.02 (2.49 ± 0.05 *µ*M)	5.97 ± 0.97 (16.2 ± 2.6 *µ*M)

Values are expressed as mean ± SD (n = 3). Different letters indicate statistically significant differences (p < 0.05, Tukey test). IC50 represents the half-maximal inhibitory concentration; AChE, acetylcholinesterase; BChE, butyrylcholinesterase.

**Table 3:** Enzyme kinetics of the extracts on AChE activity.

Samples	Concentration	Vmax (mM/min)	km (mM)	Mode of inhibition
Control	—	12.4 ± 1	10.4 ± 2	—

Galantamine	0.01 mg/mL	12.6 ± 2	16.2 ± 5	Competitive

*Blumea lacera*	0.50 mg/mL	8.35 ± 4	8.7 ± 4	Uncompetitive
	0.25 mg/mL	10.4 ± 1	8.0 ± 2	Uncompetitive
	0.10 mg/mL	10.1 ± 1	5.27 ± 1	Uncompetitive

*Byttneria pilosa*	1.00 mg/mL	5.23 ± 1	2.57 ± 1	Uncompetitive
	0.25 mg/mL	8.52 ± 2	5.54 ± 3	Uncompetitive

*Cyclea barbata*	0.50 mg/mL	14.6 ± 4	14.7 ± 7	Competitive
	0.10 mg/mL	16.3 ± 4	17.8 ± 8	Ambiguous

*Smilax guianensis*	0.50 mg/mL	6.22 ± 1	3.75 ± 2	Uncompetitive
	0.25 mg/mL	9.77 ± 1	6.70 ± 2	Uncompetitive

**Table 4:** Binding affinity of the selected isolates of *Blumea lacera* with AChE (PDB ID: 4EY7) and BChE (PDB ID: 4AQD) enzymes.

Compound name	Molecular formula	Molecular weight	Binding energy (kcal/mol)	
			AChE	BChE
5,4′-dihydroxy 6,7,3′-trimethoxyflavone (BL1)	C_18_H_16_O_7_	344.31	−9.4	−8.5
3,5,4′-trihydroxy 6,7,3′-trimethoxyflavone (BL2)	C_18_H_16_O_8_	360.31	−9.4	−8.8
5,3′,4′-trihydroxy 3,6,7-trimethoxyflavone (BL3)	C_18_H_16_O_8_	360.31	−6.8	−8.3
5-hydroxy 3,6,7,3′,4′-pentamethoxyflavone (BL4)	C_20_H_20_O_8_	388.36	−8.7	−7.7
Kaempferol-3-*O*-*α*-L-rhamnopyranosyl-(1→6)-*β*-D-glucopyranoside (BL5)	C_27_H_30_O_15_	594.51	−8.7	−10.4
Kaempferol-3-*O*-(2″,6″-di-*O*-*α*-L-rhamnopyranosyl)-*β*-D-glucopyranoside (BL6)	C_33_H_40_O_19_	740.65	−8.3	−10.7
Campesterol (BL7)	C_28_H_48_O	400.68	−10.9	−8.8
19*α*-Hydroxyurs-12-ene-24,28-dioate 3-*O*-*β*-D-xylopyranoside (BL8)	C_37_H_58_O_10_	662.85	−8.7	−10.4
(25R)-3*β*-{*O*-*β*-D-Glucopyranosyl-(1→4)-*O*-*α*-L-rhamnopyranosyl-(1→4)-[*O*-*α*-L-rhamnopyranosyl-(1→2)]-*α*-L-rhamnopyranosyl}-22*α*N-spirosol-5-ene (BL9)	C_51_H_83_NO_19_	1014.20	−8.7	−9.9
Galantamine	C_17_H_21_NO_3_	287.35	−9.4	−8.5

## Data Availability

The data used to support the findings of this study are available from the corresponding author upon request.

## Abbreviations

AD: Alzheimer's disease
AChE: Acetylcholinesterase
APP: Amyloid precursor protein
ATCI: Acetylthiocholine iodide
BChE: Butyrylcholinesterase
BSA: Bovine serum albumin
BTCI: Butyrylthiocholine iodide
CNS: Central nervous system
DTNB: 5, 5′-Dithio-bis-(2-nitrobenzoic acid).

## Supplementary Materials

Supplementary Materials Figure S1-1 shows details of collection and identification of *Blumea lacera* (Burm.f.) DC.; Figure S1-2 shows details of collection and identification of *Byttneria pilosa* Roxb.; Figure S1-3 shows details of collection and identification of *Clerodendrum infortunatum* L.; Figure S1-4 shows details of collection and identification of *Cyclea barbata* Miers; Figure S1-5 shows details of collection and identification of *Mikania micrantha* Kunth; Figure S1-6 shows details of collection and identification of *Smilax guianensis* Vitman; Figure S1-7 shows details of collection and identification of *Spermacoce articularis* L.f.; Figure S1-8 shows details of collection and identification of *Thunbergia grandiflora* Roxb.; Figure S2-1 shows the best rank pose (2D and 3D) of noncovalent interactions between ligand and AChE; Figure S2-2 shows the best rank pose (2D and 3D) of noncovalent interactions between ligand and AChE; Figure S3-1 shows the best rank pose (2D and 3D) of noncovalent interactions between ligand and BChE; Figure S3-2 shows the best rank pose (2D and 3D) of noncovalent interactions between ligand and BChE; Table S1 shows noncovalent interactions of BL-1, BL-4, and BL-7 with AChE; Table S2 shows noncovalent interactions of BL-5, BL-6, and BL-8 with BChE.
