# Supplementary material for: Anticholinesterase Activity of Eight Medicinal Plant Species: In Vitro and In Silico Studies in the Search for Therapeutic Agents against Alzheimer's Disease
Source: Evid Based Complement Alternat Med. 2021 Jun 25;2021:9995614. doi: 10.1155/2021/9995614 (PMC8260289; doi:10.1155/2021/9995614)
Supplement: Supplementary Materials — Figure S1-1 shows details of collection and identification of Blumea lacera (Burm.f.) DC.; Figure S1-2 shows details of collection and identification of Byttneria pilosa Roxb.; Figure S1-3 shows details of collection and identification of Clerodendrum infortunatum L.; Figure S1-4 shows details of collection and identification of Cyclea barbata Miers; Figure S1-5 shows details of collection and identification of Mikania micrantha Kunth; Figure S1-6 shows details of collection and identification of Smilax guianensis Vitman; Figure S1-7 shows details of collection and identification of Spermacoce articularis L.f.; Figure S1-8 shows details of collection and identification of Thunbergia grandiflora Roxb.; Figure S2-1 shows the best rank pose (2D and 3D) of noncovalent interactions between ligand and AChE; Figure S2-2 shows the best rank pose (2D and 3D) of noncovalent interactions between ligand and AChE; Figure S3-1 shows the best rank pose (2D and 3D) of noncovalent interactions between ligand and BChE; Figure S3-2 shows the best rank pose (2D and 3D) of noncovalent interactions between ligand and BChE; Table S1 shows noncovalent interactions of BL-1, BL-4, and BL-7 with AChE; Table S2 shows noncovalent interactions of BL-5, BL-6, and BL-8 with BChE. [file 9995614.f1.docx]

SUPPEMENTARY MATERIAL

**Anticholinesterase activity of eight medicinal plant species: *in vitro* and *in silico* studies in the search for therapeutic agents against Alzheimer’s disease**

Md. Josim Uddin^1^, Daniela Russo^2,3^, Md. Mahbubur Rahman^4^, Shaikh Bokhtear Uddin^5^, Mohammad A. Halim^6^, Christian Zidorn^1^, Luigi Milella^2,*^

^1^Pharmazeutisches Institut, Abteilung Pharmazeutische Biologie, Christian-Albrechts- Universität zu Kiel, Gutenbergstraße 76, 24118 Kiel, Germany

^2^Department of Science, University of Basilicata, Viale dell’ Ateneo Lucano 10, 85100 Potenza, Italy

^3^Spinoff BioActiPlant s.r.l., Department of Science, University of Basilicata, Potenza, Italy

^4^Division of infectious diseases and division of computer-aided drug design, The Red-Green Research Centre, BICCB, Tejgaon, Dhaka, Bangladesh

^5^Department of Botany, University of Chittagong, Chattogram 4331, Bangladesh

^6^Department of Physical Sciences, University of Arkansas-Fort Smith, Fort Smith, Arkansas, USA

| **List of contents** |  |
| --- | --- |
| **Content** | **Page no.** |
| Figure S1-1. Details of collection and identification of *Blumea lacera* (Burm.f.) DC. | 3 |
| Figure S1-2. Details of collection and identification of *Byttneria pilosa* Roxb. | 4 |
| Figure S1-3. Details of collection and identification of *Clerodendrum infortunatum* L. | 5 |
| Figure S1-4. Details of collection and identification of *Cyclea barbata* Miers | 6 |
| Figure S1-5. Details of collection and identification of *Mikania micrantha* Kunth | 7 |
| Figure S1-6. Details of collection and identification of *Smilax guianensis* Vitman | 8 |
| Figure S1-7. Details of collection and identification of *Spermacoce articularis* L.f. | 9 |
| Figure S1-8. Details of collection and identification of *Thunbergia grandiflora* Roxb. | 10 |
| Figure S2-1. The best rank pose (2D and 3D) of non-covalent interactions between ligand and AChE | 11 |
| Figure S2-2. The best rank pose (2D and 3D) of non-covalent interactions between ligand and AChE | 12 |
| Figure S3-1. The best rank pose (2D and 3D) of non-covalent interactions between ligand and BChE | 13 |
| Figure S3-2. The best rank pose (2D and 3D) of non-covalent interactions between ligand and BChE | 14 |
| Table S1. Noncovalent interactions of BL-1, BL-4, and BL-7 with AChE | 15-16 |
| Table S2. Noncovalent interactions of BL-5, BL-6, and BL-8 with BChE | 17-18 |

**
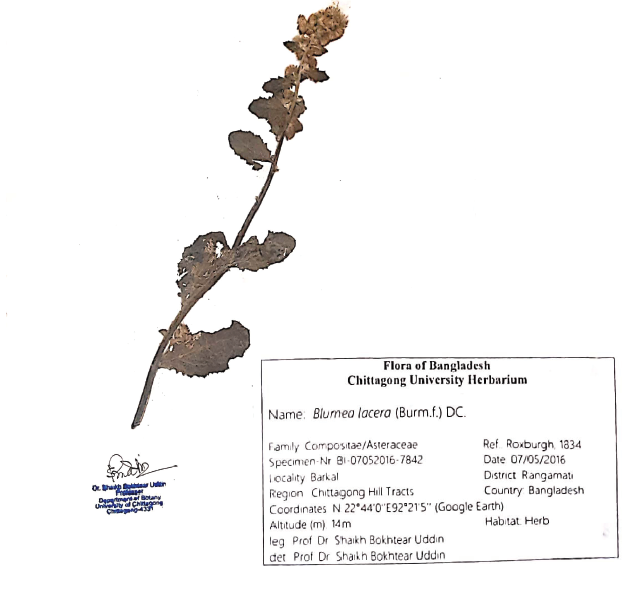
**

Figure S1-1

Figure S1-1


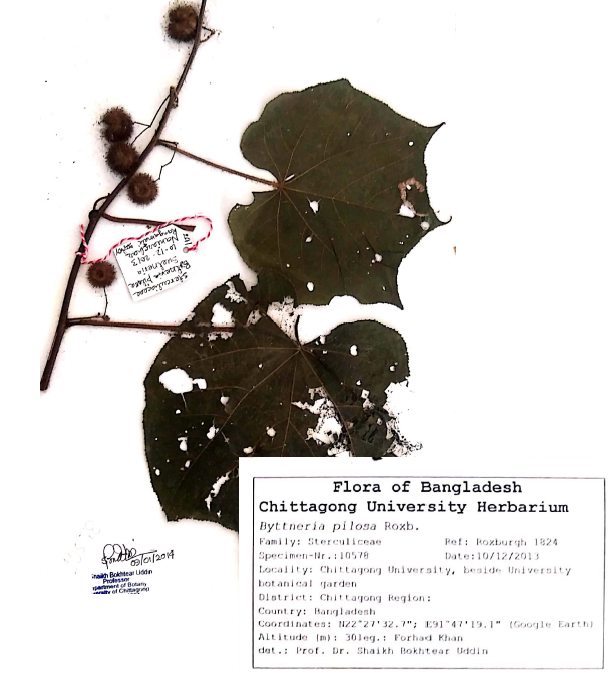


Figure S1-2


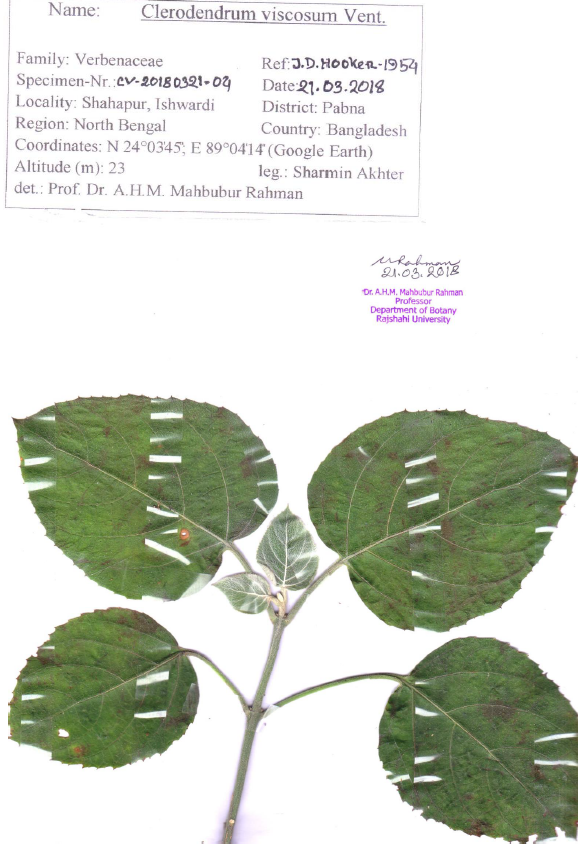


Figure S1-3


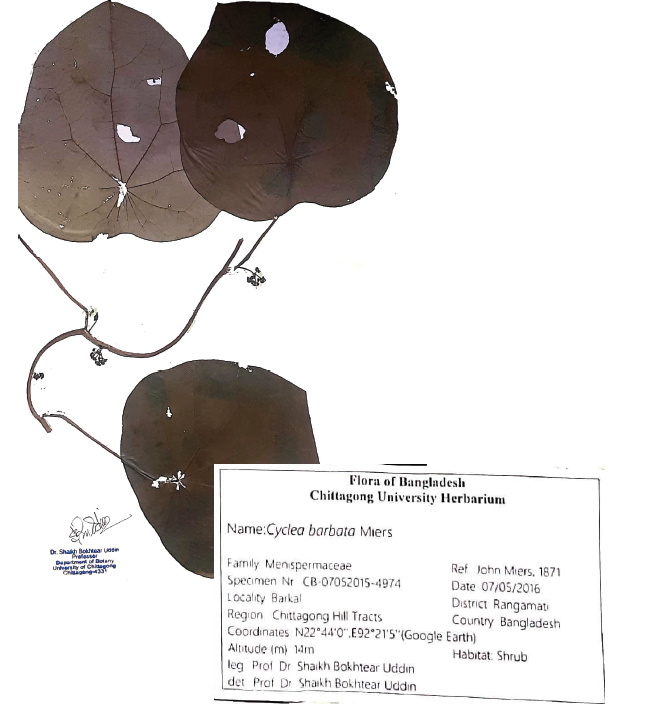


Figure S1-4


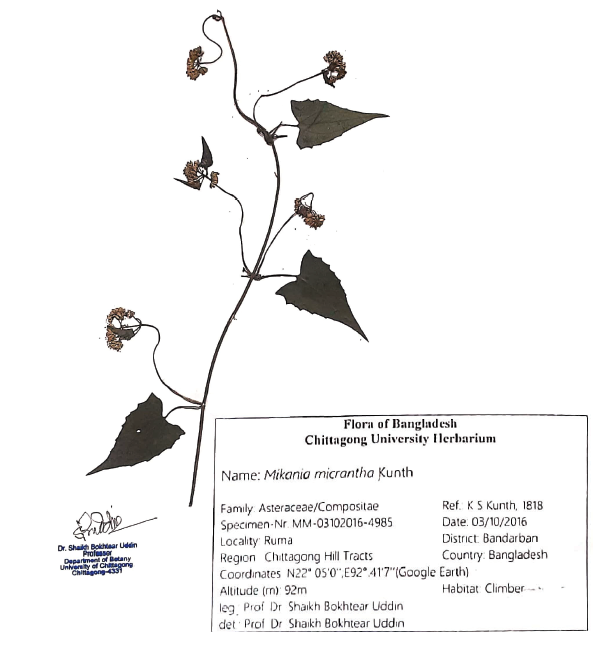


Figure S1-5


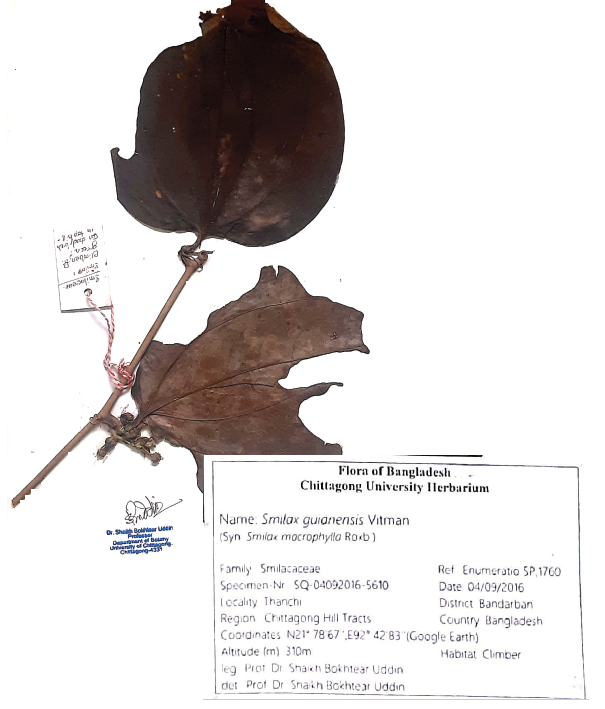


Figure S1-6


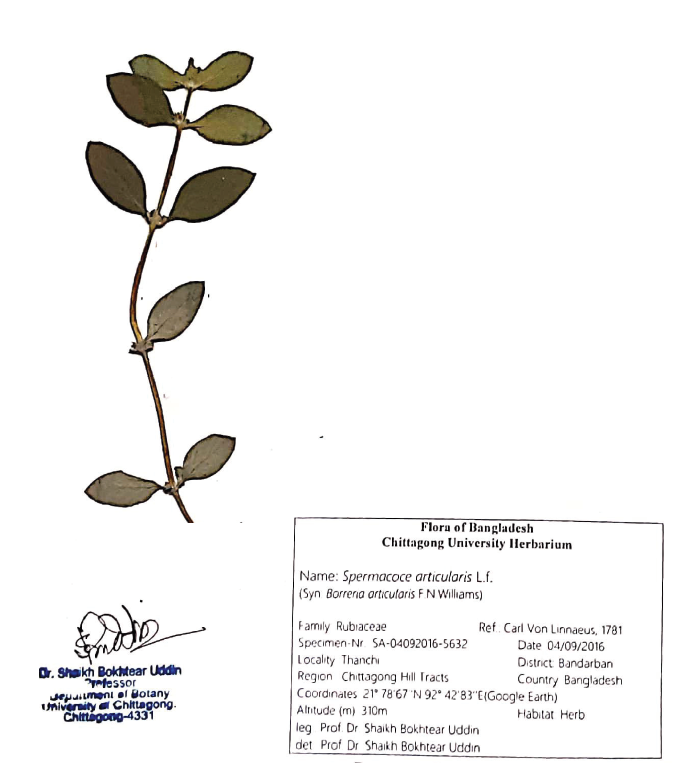


Figure S1-7


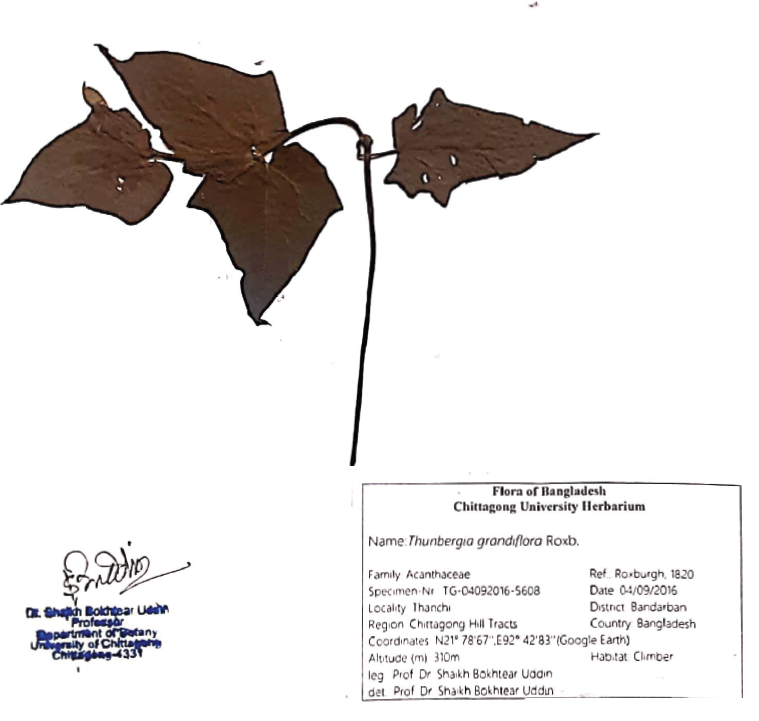


Figure S1-8


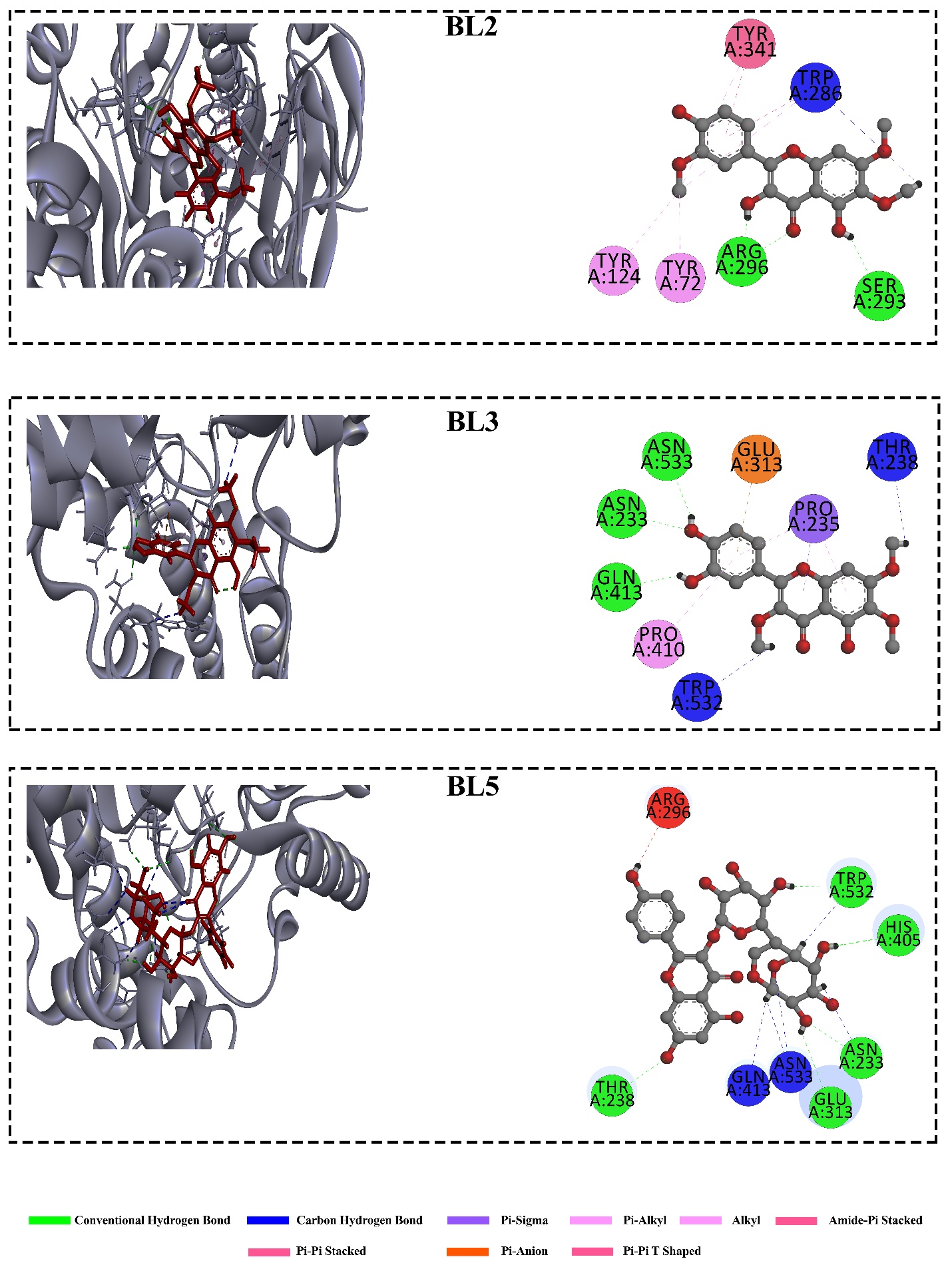
**Fig. S2-1**: The best rank pose (2D and 3D) of non-covalent interactions between ligand and AChE (PDB-ID 4EY7). In 3D, ligand molecules have been represented as red, and proteins are dark silver (pose predicted by AutoDock Vina).


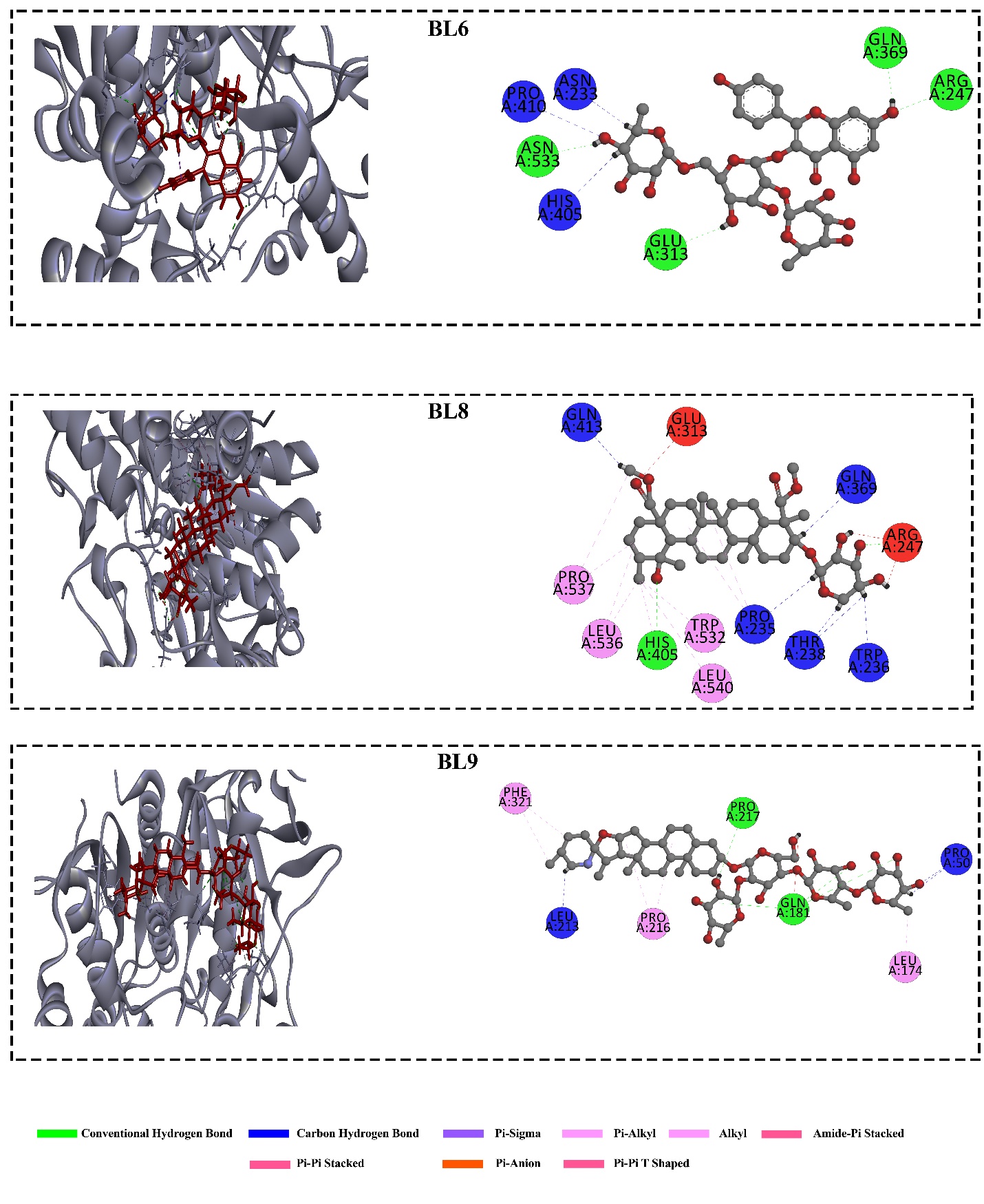
**Fig. S2-2**: The best rank pose (2D and 3D) of non-covalent interactions between ligand and AChE (PDB-ID 4EY7). In 3D, ligand molecules have been represented as red, and proteins are dark silver (pose predicted by AutoDock Vina).


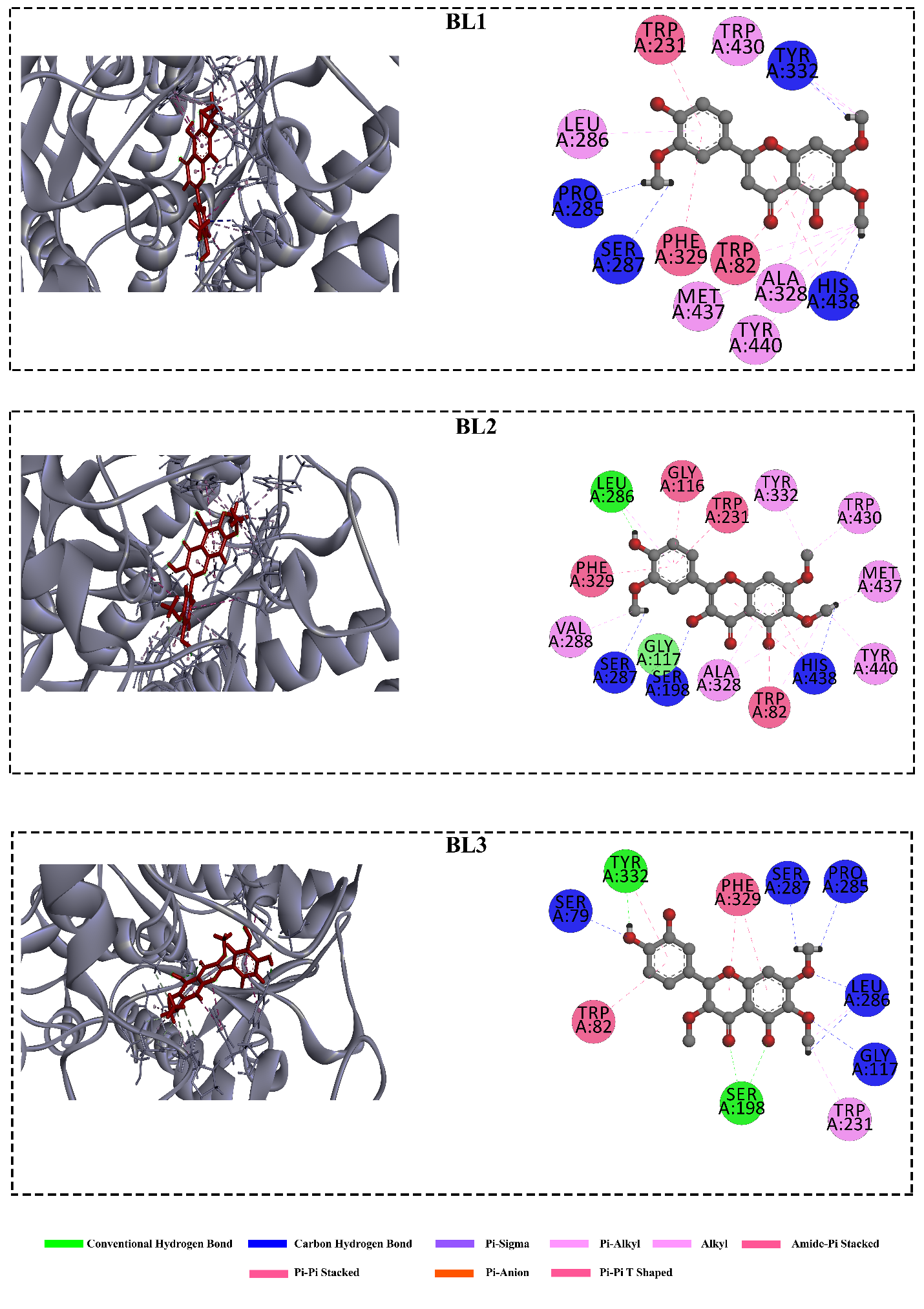
**Fig. S3-1**: The best rank pose (2D and 3D) of non-covalent interactions between ligand and BChE (PDB-ID 4AQD). In 3D, ligand molecules have been represented as red, and proteins are dark silver (pose predicted by AutoDock Vina).


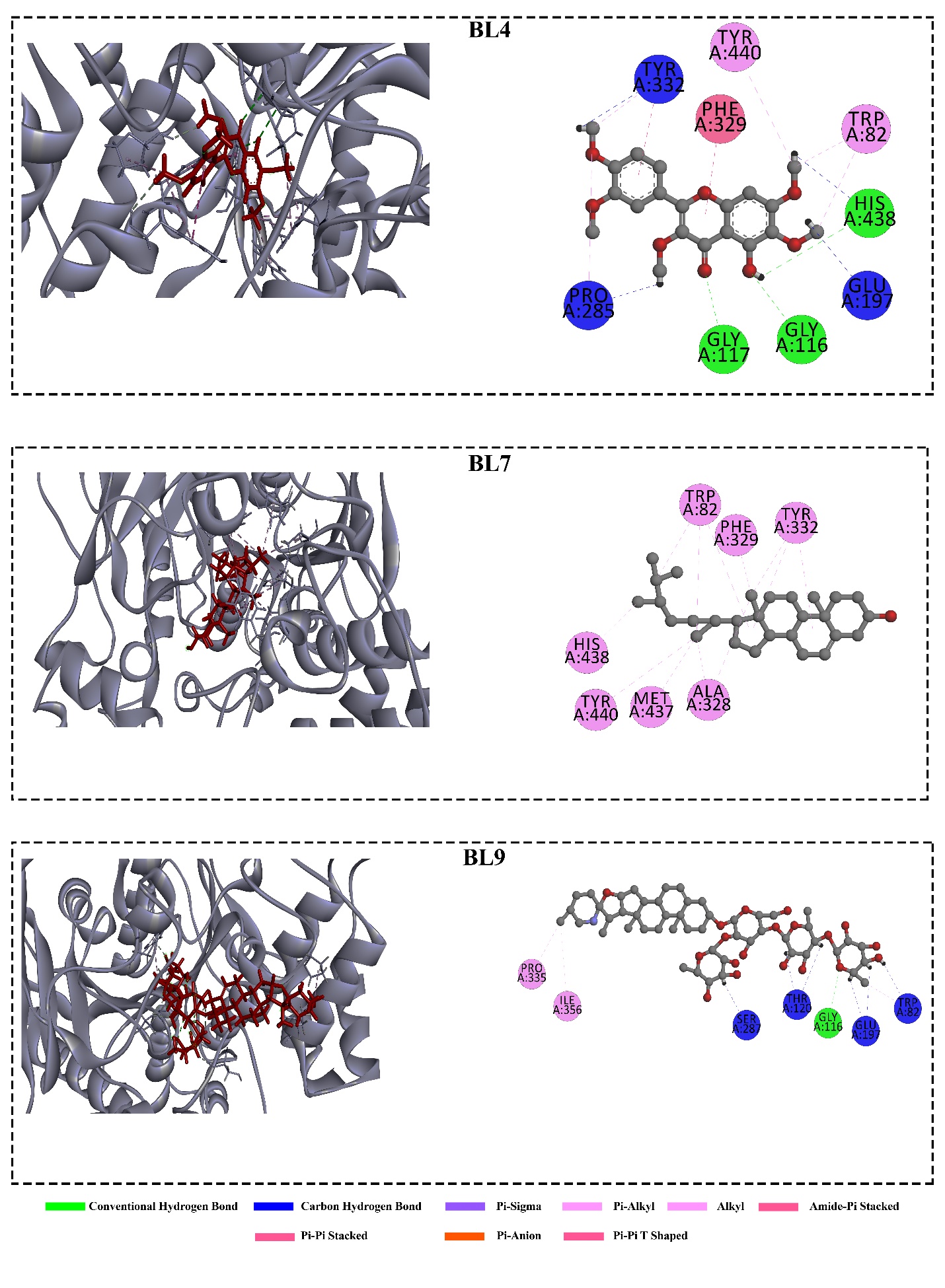
**Fig. S3-2**: The best rank pose (2D and 3D) of non-covalent interactions between ligand and BChE (PDB-ID 4AQD). In 3D, ligand molecules have been represented as red, and proteins are dark silver (pose predicted by AutoDock Vina).

**Table S1:** Noncovalent interactions of BL-1, BL-4, and BL-7 with AChE (pose predicted by AutoDock Vina)

| **Compound** | **Residue** | **Distance (Å)** | **Type** | **Category** |
| --- | --- | --- | --- | --- |
| **BL1** | Thr83 | 3.01 | Hydrogen Bond | Carbon Hydrogen Bond |
|  | Asp74 | 2.251 | Hydrogen Bond | Carbon Hydrogen Bond |
|  | Tyr124 | 2.194 | Hydrogen Bond | Pi-Donor Hydrogen Bond |
|  | Trp296 | 4.999 | Hydrophobic | Pi-Alkyl |
|  | Tyr341 | 4.512 | Hydrophobic | Pi-Alkyl |
|  | Tyr124 | 5.334 | Hydrophobic | Pi-Alkyl |
|  | Phe338 | 4.902 | Hydrophobic | Pi-Alkyl |
|  | Tyr341 | 5.356 | Hydrophobic | Pi-Alkyl |
|  | Trp86 | 5.699 | Hydrophobic | Pi-Alkyl |
|  | Tyr72 | 4.855 | Hydrophobic | Pi-Alkyl |
|  | Trp86 | 4.160 | Hydrophobic | Pi-Alkyl |
|  | Trp86 | 3.857 | Hydrophobic | Pi-Alkyl |
|  | Trp86 | 3.889 | Hydrophobic | Pi-Alkyl |
|  | Trp286 | 4.457 | Hydrophobic | Pi-Alkyl |
|  | Trp286 | 3.969 | Hydrophobic | Pi-Alkyl |
|  | Tyr337 | 5.319 | Hydrophobic | Pi-Alkyl |
|  | Tyr337 | 4.345 | Hydrophobic | Pi-Alkyl |
|  | Tyr341 | 5.102 | Hydrophobic | Pi-Alkyl |
|  | His447 | 5.498 | Hydrophobic | Pi-Alkyl |
|  | | | | |
| **BL4** | Gly121 | 2.745 | Hydrogen bond | Conventional hydrogen bond |
|  | Gly122 | 2.502 | Hydrogen bond | Conventional hydrogen bond |
|  | Ser203 | 2.344 | Hydrogen bond | Conventional hydrogen bond |
|  | Ser203 | 1.943 | Hydrogen bond | Conventional hydrogen bond |
|  | Ser293 | 2.519 | Hydrogen bond | Carbon hydrogen bond |
|  | Arg296 | 3.092 | Hydrogen bond | Carbon hydrogen bond |
|  | Asp74 | 1.846 | Hydrogen bond | Carbon hydrogen bond |
|  | Tyr124 | 2.82 | Hydrogen bond | Carbon hydrogen bond |
|  | Tyr124 | 2.646 | Hydrogen bond | Carbon hydrogen bond |
|  | Glu202 | 2.361 | Hydrogen bond | Carbon hydrogen bond |
|  | Tyr124 | 2.856 | Other | Pi-Lone pair |
|  | Trp286 | 5.234 | Hydrophobic | Pi-Pi stacked |
|  | Tyr341 | 4.52 | Hydrophobic | Pi-Pi stacked |
|  | Tyr124 | 5.191 | Hydrophobic | Pi-Pi T-shaped |
|  | Tyr341 | 5.794 | Hydrophobic | Pi-Pi T-shaped |
|  | Tyr124 | 5.801 | Hydrophobic | Pi-Pi T-shaped |
|  | Phe297 | 5.798 | Hydrophobic | Pi-Pi T-shaped |
|  | Tyr72 | 5.085 | Hydrophobic | Pi-Alkyl |
|  | Trp236 | 4.479 | Hydrophobic | Pi-Alkyl |
|  | Trp286 | 4.564 | Hydrophobic | Pi-Alkyl |
|  | Trp286 | 3.884 | Hydrophobic | Pi-Alkyl |
|  | Trp286 | 4.842 | Hydrophobic | Pi-Alkyl |
|  | Phe295 | 4.524 | Hydrophobic | Pi-Alkyl |
|  | Phe297 | 4.661 | Hydrophobic | Pi-Alkyl |
|  | Tyr341 | 4.937 | Hydrophobic | Pi-Alkyl |
|  | His447 | 4.892 | Hydrophobic | Pi-Alkyl |
|  | His447 | 4.208 | Hydrophobic | Pi-Alkyl |
|  | | | | |
| **BL7** | Glu202 | 2.987 | Hydrogen bond | Conventional hydrogen bond |
|  | Tyr124 | 5.453 | Hydrophobic | Pi-Alkyl |
|  | Tyr124 | 4.98 | Hydrophobic | Pi-Alkyl |
|  | Trp286 | 4.148 | Hydrophobic | Pi-Alkyl |
|  | Trp286 | 4.684 | Hydrophobic | Pi-Alkyl |
|  | Trp286 | 3.514 | Hydrophobic | Pi-Alkyl |
|  | Phe297 | 4.457 | Hydrophobic | Pi-Alkyl |
|  | Tyr337 | 4.58 | Hydrophobic | Pi-Alkyl |
|  | Phe338 | 4.484 | Hydrophobic | Pi-Alkyl |
|  | Phe338 | 4.648 | Hydrophobic | Pi-Alkyl |
|  | Tyr341 | 3.642 | Hydrophobic | Pi-Alkyl |
|  | Tyr341 | 5.177 | Hydrophobic | Pi-Alkyl |
|  | His447 | 4.812 | Hydrophobic | Pi-Alkyl |

**Table S2.** Noncovalent interactions of BL-5, BL-6, and BL-8 with BChE (pose predicted by AutoDock Vina)

| **Compound** | **Residue** | **Distance (Å)** | **Type** | **Category** |
| --- | --- | --- | --- | --- |
| **BL5** | Leu286 | 1.873 | Hydrogen | Conventional hydrogen bond |
|  | His438 | 2.543 | Hydrogen | Carbon hydrogen bond |
|  | Glu197 | 2.774 | Hydrogen | Carbon hydrogen bond |
|  | His438 | 3.028 | Hydrogen | Carbon hydrogen bond |
|  | Trp82 | 2.707 | Hydrogen | Carbon hydrogen bond |
|  | Gly116 | 2.2 | Hydrogen | Carbon hydrogen bond |
|  | Gly116 | 2.81 | Hydrogen | Carbon hydrogen bond |
|  | Ser198 | 3.071 | Hydrogen | Carbon hydrogen bond |
|  | His438 | 1.843 | Hydrogen | Carbon hydrogen bond |
|  | Gly116 | 4.734 | Hydrophobic | Amide-pi stacked |
|  | Leu286 | 5.413 | Hydrophobic | Pi-alkyl |
|  | | | | |
| **BL6** | Asn83 | 2.185 | Hydrogen bond | Conventional hydrogen bond |
|  | Glu197 | 2.08 | Hydrogen bond | Conventional hydrogen bond |
|  | His438 | 2.261 | Hydrogen bond | Conventional hydrogen bond |
|  | His438 | 2.84 | Hydrogen bond | Conventional hydrogen bond |
|  | Asp70 | 2.428 | Hydrogen bond | Conventional hydrogen bond |
|  | Tyr332 | 3.059 | Hydrogen bond | Carbon hydrogen bond |
|  | His438 | 2.784 | Hydrogen bond | Carbon hydrogen bond |
|  | Gly116 | 2.521 | Hydrophobic | Pi-sigma |
|  | Trp82 | 5.228 | Hydrophobic | Pi-pi t-shaped |
|  | Gly115 | 3.774 | Hydrophobic | Amide-pi stacked |
|  | | | | |
| **BL8** | Ala277 | 2.808 | Hydrogen bond | Conventional hydrogen bond |
|  | Asn289 | 2.307 | Hydrogen bond | Conventional hydrogen bond |
|  | Ser287 | 2.758 | Hydrogen bond | Carbon hydrogen bond |
|  | Thr284 | 2.93 | Hydrogen bond | Carbon hydrogen bond |
|  | Ala328 | 2.563 | Hydrogen bond | Carbon hydrogen bond |
|  | Ala277 | 2.67 | Hydrogen bond | Carbon hydrogen bond |
|  | Ala328 | 3.845 | Hydrophobic | Alkyl |
|  | Ala277 | 4.307 | Hydrophobic | Alkyl |
|  | Ala328 | 3.357 | Hydrophobic | Alkyl |
|  | Trp82 | 4.468 | Hydrophobic | Pi-Alkyl |
|  | Trp82 | 5.262 | Hydrophobic | Pi-Alkyl |
|  | Phe329 | 4.738 | Hydrophobic | Pi-Alkyl |
|  | Tyr332 | 4.043 | Hydrophobic | Pi-Alkyl |
|  | Tyr332 | 5.007 | Hydrophobic | Pi-Alkyl |
|  | Trp430 | 4.696 | Hydrophobic | Pi-Alkyl |
|  | Trp430 | 4.017 | Hydrophobic | Pi-Alkyl |
|  | His438 | 4.434 | Hydrophobic | Pi-Alkyl |
